# The burden of headache disorders among adults in Peru: national estimates and a health-care needs assessment from a population-based door-to-door survey

**DOI:** 10.1186/s10194-024-01902-3

**Published:** 2024-11-28

**Authors:** Guiovanna Quispe, Cesar Loza, Luis Limaco, Ruth Gallegos, Carlos Palomino-Diaz, Ivett Cruz, Jacqueline Miranda, Liliana Rodriguez, Andreas Husøy, Timothy J. Steiner

**Affiliations:** 1 Neurology Service, Hospital Luis Negreiros Vega, Callao, Peru; 2 Department of Nephrology, Peruvian University Cayetano Heredia, Lima, Peru; 3 National Institute of Statistics and Informatics, Lima, Peru; 4 Neurology Service, Hospital Daniel Alcides Carrion, Callao, Peru; 5 Neurology Service, Hospital Nacional Edgardo Rebagliati Martins, Lima, Peru; 6 NorHEAD, Department of Neuromedicine and Movement Science, Norwegian University of Science and Technology (NTNU), Edvard Griegs gate, Trondheim, Norway; 7 Department of Neurology, University of Copenhagen, Copenhagen, Denmark; 8 Division of Brain Sciences, Imperial College London, London, UK

**Keywords:** Headache disorders, Migraine, Tension-type headache, Medication-overuse headache, Epidemiology, Burden of disease, Population-based survey, Peru, South America, Global Campaign against Headache

## Abstract

**Background:**

We have previously found that almost two thirds (64.6%) of adults in Peru have an active headache disorder. Here, using data from the same sample, we present attributed burden at individual and population levels. We use these data to assess need for headache-related health care among this population.

**Methods:**

We used the standard methodology of the Global Campaign against Headache. Cluster-sampling from five geographical regions of Peru (Cajamarca, Lima, Piura, Puno, San Martín) generated a sample representative of the population aged 18–65 years. At unannounced visits to households, we interviewed one adult from each using the HARDSHIP questionnaire. We assessed symptom burden in terms of headache frequency and usual duration and intensity, and impaired participation in paid work, household work and social or leisure activities using the HALT index. To assess need for health care, we counted all those with headache on ≥ 15 days/month (H15+), those with migraine on ≥ 3 days/month, and those with migraine or tension-type headache meeting either of two criteria: a) proportion of time in ictal state (pTIS) > 3.3% *and* intensity ≥ 2 (moderate-to-severe); b) ≥ 3 lost days from paid and/or household work during the preceding 3 months. We derived population-level estimates by factoring in prevalences.

**Results:**

The sample size was *N* = 2,149. From individual data, we estimated population-level pTIS at 1.9–2.5%, this proportion of all time among adults in Peru being spent with headache, with migraine the greatest contributor (1.2%). At population level, headache was responsible for 0.5 days lost from paid work and 1.0 days from household work per person per 3 months, with migraine again the biggest contributor (0.2 and 0.5 days). However, at individual level, H15 + was associated with greatest burden (pTIS 14.9–24.9%; 2.3–4.5 lost workdays/3 months). A quarter of the sample (*n* = 590; 27.5%) fulfilled one or more of our health-care need criteria.

**Conclusion:**

Headache disorders are responsible for high levels of ill health and economic burden in Peru, with a substantial requirement for health care. Health and economic policies balancing health benefits against the cost of providing care should take account of the productivity losses that might be recovered, and the expected cost-offset.

## Background

The Global Campaign against Headache, conducted in collaboration with the World Health Organization, has been in progress for over 20 years [1]. Its first objective has been to fill the large geographical gaps that existed in knowledge of the global burden of headache [2], requiring population-based studies using standard consensus-based methodology [3, 4]. Such studies have now been conducted in countries in all world regions [5–17], informing not only our understanding of the global headache burden but also regional and national health policies, priority setting and best allocation of health-care resources.

This study, in Peru – the first in South America – was one in this series. It has already shown headache disorders to be prevalent among adults in this country [17], with estimates for both migraine (22.8%) and tension-type headache (TTH: 38.9%) exceeding global averages [18, 19]. Here, using data collected contemporaneously in the same study, we present estimates of important components of the burden attributed to these disorders. We also conduct a needs assessment for headache-related health care.

## Methods

This was a cross-sectional population-based study employing the standardized methodology developed by the Global Campaign [3, 4]. The specific details of study design and methodology have been published previously [17].

### Study design and interview

Adopting randomized cluster-sampling, we selected five geographical regions of Peru (Cajamarca, Lima, Piura, Puno and San Martín), urban and rural communities within each, and individual households in each community. Since only 22% of the Peruvian population resided in non-urban regions, we oversampled rural areas to achieve sufficient statistical power for urban/rural comparisons. During May to November 2019, trained health workers made unannounced visits to each household and interviewed one randomly selected adult inhabitant (aged 18–65 years) using the structured Headache-Attributed Restriction, Disability, Social Handicap and Impaired Participation (HARDSHIP) questionnaire [4]. Demographic enquiry and headache diagnostic questions based on ICHD-3 [20] were followed by enquiry into symptom burden, impaired participation in daily life, willingness to pay (WTP) for effective headache treatment and impact of headache on quality of life (QoL). Participants reporting headaches of more than one type were asked to focus on whichever they felt was the most bothersome.

Separate enquiry included symptoms and headache-attributed impaired participation yesterday among those reporting headache yesterday (HY).

Diagnoses were algorithmically determined. Participants reporting headache on ≥ 15 days/month (H15+) were first identified. H15 + was diagnosed as probable medication-overuse headache (pMOH) when associated with acute headache medication usage on ≥ 15 days/month and otherwise as “other H15+”. In those with episodic headache (on < 15 days/month), the algorithm applied modified ICHD-3 criteria [20] to diagnose definite migraine, definite TTH, probable migraine and probable TTH, in that order. Definite and probable migraine, and definite and probable TTH, were combined for burden analysis.

We did not attempt to diagnose HY, since ICHD criteria apply to disorders, not single episodes [20].

### Burden

We measured symptom burden in terms of frequency (reported in days/month), usual duration (in hours) and usual intensity of headache (reported as “mild”, “moderate” or “severe”, which we transformed into a numerical scale 1–3), We derived proportion of time spent in the ictal state (pTIS) as [(frequency*duration)/(24*30)], with duration capped at 24 h since frequency was reported as days/month and not attacks/month. We calculated lost health at individual level attributed to migraine, TTH or pMOH as pTIS*DW, where DW was the disability weight attached by the Global Burden of Disease (GBD) study to each disorder [21].

We assessed impaired participation due to headache over the preceding 3 months through the Headache-Attributed Lost Time (HALT) index [22], a module within HARDSHIP [4]. Separate enquiries regarded paid work, household work (the essential chores of daily life) and social or leisure activities. If data were missing we applied a value of zero on the assumption that participants were likely not to respond when an activity seemed irrelevant (for example, those who were unemployed could not lose days from paid work). In accordance with established methodology, we counted lost days in each domain, equating “less than half achieved” on a particular day with “nothing achieved” (one day lost) and, to counterbalance, “more than half achieved” with “everything achieved” (no days lost) [22]. In individuals reporting HY, overall impaired participation yesterday (not distinguishing between different domains) was similarly assessed.

We enquired into WTP, in Peruvian nuevo sol (PEN) per month, using the bidding game method [4]. At the time of the study, PEN 1.00 = USD 0.30. We measured QoL, in all participants, with or without headache, on a scale of 8–40, higher scores indicating better QoL, by summing the self-reported scores, each on a scale of 1–5, of the World Health Organization quality of life 8-item questionnaire (WHOQoL-8) [23].

### Health-care needs assessment

We assumed that need for health care existed only in those expected to benefit from it. We counted all those fulfilling one or more of the following criteria: (1) reporting H15+; (2) reporting headache diagnosed as migraine on ≥ 3 days/month; (3) diagnosed as migraine or TTH, with pTIS > 3.3% *and* moderate or severe headache; (4) diagnosed as migraine or TTH *and* reporting ≥ 3 days lost from paid or household work during the preceding 3 months.

### Statistics and analysis

We treated headache frequency, headache duration, pTIS, data derived from HALT, WTP and WHOQoL-8 scores as continuous variables, and headache intensity and participation yesterday as categorical variables. We analysed continuous variables using means, standard errors (SEMs) and medians, and made comparisons (between genders and headache types when appropriate) using ANOVA tests. We analysed categorical variables using exact figures and proportions (%), and made comparisons (between genders) using chi-square tests.

Age and gender-adjusted estimates of pTIS, lost health and impaired participation at population level were calculated by factoring in our previously published prevalence estimates [17]. Estimates based on recall were made from pTIS (a product of headache frequency and usual duration), HALT data and 1-year prevalence. Additional estimates were based on HY data (headache duration and impaired participation yesterday) and 1-day prevalence.

We performed all statistical analyses using SPSS version 28 (SPSS, INC, Chicago, IL). We set significance at *p* < 0.05. Figures were made using the ggplot package within RStudio version 2023.06.2 + 561.

## Results

There were 2,149 participants. The prevalence estimates have been published previously [17], but are summarized here because they are used in our analyses. Headache in the preceding year was reported by 64.6% (57.6% of males, 71.5% of females). Adjusted for age and gender, the 1-year prevalence of migraine was 22.8%, of TTH 38.9%, of pMOH 1.2% and of other H15 + 2.7% [17]. HY was reported by 12.1% of participants [17].

### Individual burden

**Fig. 1:**
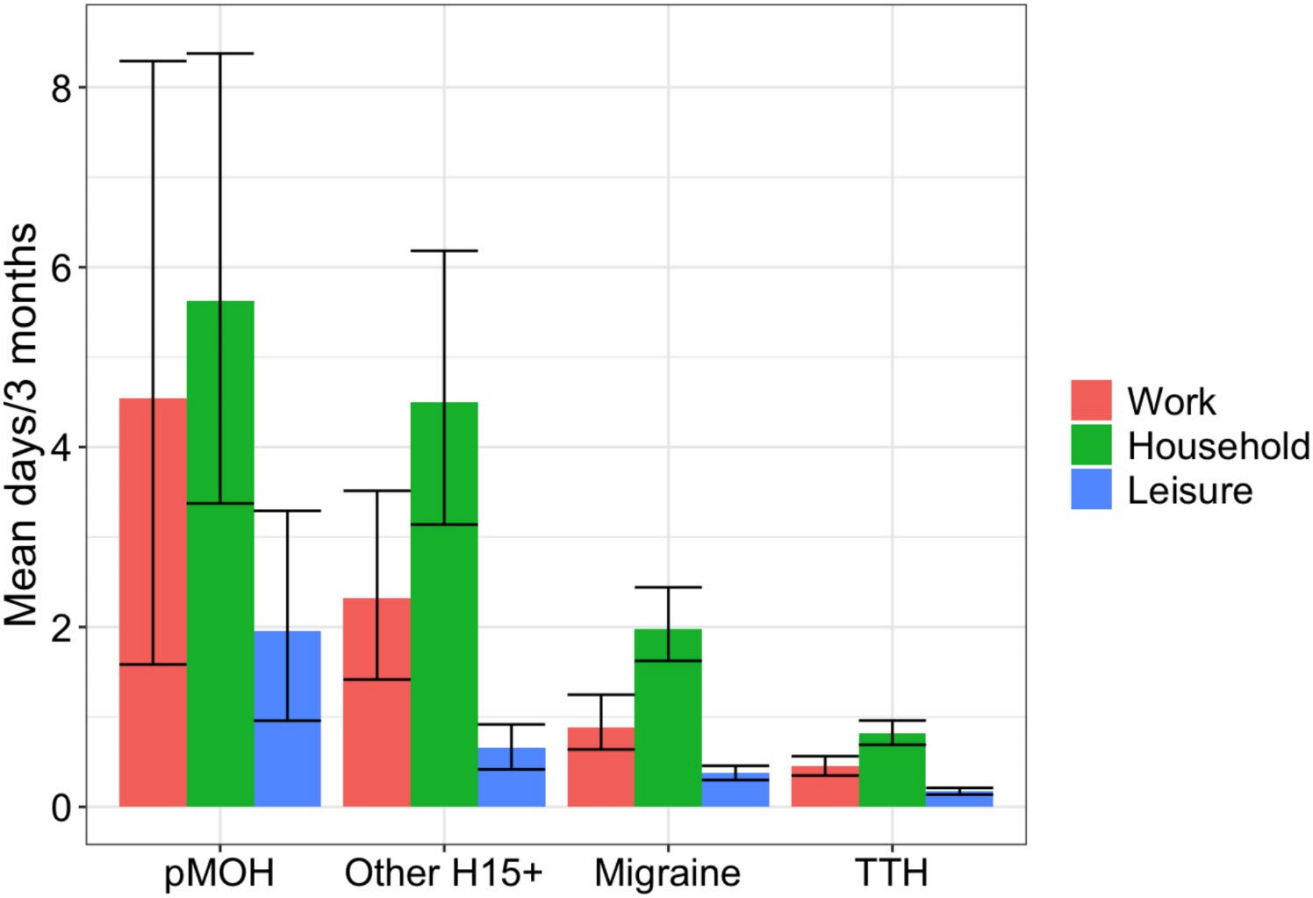
Lost days from paid work (red), household work (green) and social or leisure activities (blue) at individual level, by headache type (pMOH: probable medication-overuse headache; H15+: headache on ≥ 15 days/month; TTH: tension-type headache)

Table 1 summarizes the symptom burden, Table 2 and Fig. 1 the impaired participation attributed to all headache and to each headache type. Participants with any headache reported, on average, 4.2 days/month with headache of mean duration 7.0 h and mean intensity 1.4 (mild-to-moderate). Mean pTIS was 3.8%. Females reported significantly higher frequency (*p* = 0.009), duration (*p* < 0.001) and intensity (*p* < 0.001) than males (Table 1). As measures of impaired participation, those with any headache lost 0.8 days/3 months from paid work, 1.5 days from household work and 0.3 days from social or leisure activities. Males lost more days from paid work than females (1.1 vs. 0.5 days/3 months, *p* < 0.001), but no differences were found with regard to household work or lost social or leisure activities (Table 2).

**Table 1:** Headache-attributed symptom burden and lost health at individual level, overall and by gender

	Overall	Male	Female	Male vs. female
**Frequency** (days/month) (mean±SEM, median)				
Any headache	4.2±0.1, 3.0	3.8±0.2, 2.0	4.5±0.2, 3.0	F(1, 1386) = 6.9, ***p* = 0.009**
pMOH	22.2±1.2, 20.0	23.3±1.9, 20.0	21.6±1.7, 20.0	F(1, 22) = 0.6, *p* = 0.46
Other15+	17.8±0.9, 15.0	17.9±1.2, 16.5	16.3±1.1, 15.0	F(1, 70) = 0.2, *p* = 0.65
Migraine	3.9±0.1, 3.0	3.3±0.2, 3.0	4.2±0.2, 3.0	F(1, 479) = 9.5, ***p* = 0.002**
TTH	2.7±0.1, 2.0	2.6±0.1, 2.0	2.8±0.1, 2.0	F(1, 809) = 1.7, *p* = 0.19
**Duration** (hours) (mean±SEM, median)				
Any headache	7.0±0.3, 4.0	5.7±0.4, 3.0	8.1±0.5, 4.0	F(1, 1376) = 16.3, ***p* < 0.001**
pMOH	7.4±1.6, 4.0	8.1±3.1, 5.0	6.8±1.8, 4.0	F(1, 19) = 0.1, *p* = 0.71
Other15+	7.6±1.3, 4.0	4.5±1.0, 3.5	9.7±2.1, 6.0	F(1, 63) = 3.9, *p* = 0.05
Migraine	11.1±0.6, 6.0	8.8±0.6, 6.0	12.5±0.8, 7.0	F(1, 479) = 9.4, ***p* = 0.002**
TTH	4.6±0.3, 2.0	4.4±0.4, 2.0	4.8±0.5, 2.0	F(1, 809) = 0.3, *p* = 0.58
**Intensity** (n) (mild-moderate-severe; mean calculated by equating to 1, 2, 3)				
Any headache	898-446-44 (mean = 1.4)	433-176-13 (mean = 1.3)	465-279-31 (mean = 1.4)	*X*^*2*^(2, *N* = 1388) = 18.0, ***p* < 0.001**
pMOH	4-16-4 (mean = 2.0)	3-5-1 (mean = 1.8)	1-11-3 (mean = 2.1)	*X*^*2*^(2, *N* = 24) = 2.9, *p* = 0.23
Other15+	25-39-8 (mean = 1.8)	12-13-3 (mean = 1.8)	13-26-5 (mean = 1.8)	*X*^*2*^(2, *N* = 72) = 1.4, *p* = 0.50
Migraine	160-299-22 (mean = 1.7)	61-110-4 (mean = 1.7)	99-189-18 (mean = 1.7)	*X*^*2*^(2, *N* = 481) = 3.4, *p* = 0.19
TTH	709-92-10 (mean = 1.1)	357-39-5 (mean = 1.1)	352-53-5 (mean = 1.2)	*X*^*2*^(2, *N* = 811) = 2.1, *p* = 0.36
**Proportion of time in ictal state** (%) (mean±SEM, median)				
Any headache	3.8±0.2, 1.1	2.9±0.3, 0.8	4.6±0.3, 1.6	F(1, 1376) = 16.0, ***p* < 0.001**
pMOH	24.9±6.5, 12.5	26.6±11.3, 13.9	23.7±7.9, 10.8	F(1, 19) = 0.0, *p* = 0.83
Other15+	14.9±2.2, 8.3	11.8±2.8, 7.7	17.0±3.1, 8.9	F(1, 63) = 1.3, *p* = 0.24
Migraine	5.3±0.3, 3.3	3.7±0.3, 2.7	6.2±0.4, 3.3	F(1, 479) = 15.3, ***p* < 0.001**
TTH	1.5±0.1, 0.5	1.4±0.1, 0.5	1.7±0.2, 0.5	F(1, 809) = 1.9, *p* = 0.17
**Lost health** (%) (mean±SEM, median)				
pMOH	5.6±1.4, 2.8	5.9±2.5, 3.1	5.3±1.8, 2.4	F(1, 19) = 0.0, *p* = 0.83
Migraine	2.3±0.1, 1.4	1.6±0.1, 1.2	2.7±0.2, 1.4	F(1, 479) = 15.3, ***p* < 0.001**
TTH	0.1±0.0, 0.0	0.1±0.0, 0.0	0.1±0.0, 0.0	F(1, 809) = 1.9, *p* = 0.17

pMOH: probable medication-overuse headache; H15+: headache on ≥ 15 days/month; TTH: tension-type headache; significant p-values (< 0.05) are emboldened

**Table 2:** Headache-attributed impaired participation in paid and household work and in social or leisure activities at individual level, overall and by gender

	Overall	Male	Female	Male vs. female
	Lost days/3 months Mean±SEM, median			
**Paid work (HALT questions 1 + 2)**				
Any headache	0.8±0.1, 0.0	1.1±0.2, 0.0	0.5±0.1, 0.0	F(1, 1386) = 19.3, ***p* < 0.001**
pMOH	4.5±1.7, 1.5	10.2±4.0, 4.0	1.1±0.5, 0.0	F(1, 22) = 8.5, ***p* = 0.01**
Other15+	2.3±0.5, 1.0	4.0±1.3, 3.0	1.2±0.3, 0.0	F(1, 70) = 6.4, ***p* = 0.01**
Migraine	0.9±0.2, 0.0	1.3±0.4, 0.0	0.7±0.1, 0.0	F(1, 479) = 3.2, *p* = 0.07
TTH	0.5±0.1, 0.0	0.7±0.1, 0.0	0.2±0.0, 0.0	F(1, 809) = 21,5, ***p* < 0.001**
	F(3, 1384) = 25.7, ***p* < 0.001**			
**Household work (HALT questions 3 + 4)**				
Any headache	1.5±0.1, 0.0	1.3±0.2, 0.0	1.7±0.1, 0.0	F(1, 1386) = 3.6, *p* = 0.06
pMOH	5.6±1.3, 4.5	6.4±3.4, 3.0	5.1±0.8, 5.0	F(1, 22) = 0.2, *p* = 0.64
Other15+	4.5±0.8, 3.0	4.1±1.7, 1.5	4.7±0.7, 4.5	F(1, 70) = 0.1, *p* = 0.72
Migraine	2.0±0.2, 0.0	1.9±0.5, 0.0	2.0±0.2, 0.0	F(1, 479) = 0.1, *p* = 0.80
TTH	0.8±0.1, 0.0	0.7±0.1, 0.0	0.9±0.1, 0.0	F(1, 809) = 3.2, *p* = 0.08
	F(3, 1384) = 40.3, ***p* < 0.001**			
**Social or leisure activities (HALT question 5)**				
Any headache	0.3±0.0, 0.0	0.3±0.0, 0.0	0.3±0.0, 0.0	F(1, 1386) = 0.66, *p* = 0.42
pMOH	2.0±0.6, 1.0	3.3±1.6, 1.0	1.1±0.3, 0.0	F(1, 22) = 3.2, *p* = 0.09
Other15+	0.7±0.1, 0.0	0.6±0.2, 0.0	0.7±0.2, 0.0	F(1, 70) = 0.0, *p* = 0.95
Migraine	0.4±0.0, 0.0	0.5±0.1, 0.0	0.3±0.0, 0.0	F(1, 479) = 4.5, ***p* = 0.03**
TTH	0.2±0.0, 0.0	0.2±0.0, 0.0	0.2±0.0, 0.0	F(1, 809) = 1.0, *p* = 0.33
	F(3, 1384) = 42.4, ***p* < 0.001**			

HALT: Headache-Attributed Lost Time index; pMOH: probable medication-overuse headache; H15+: headache on ≥ 15 days/month; TTH: tension-type headache; significant p-values (< 0.05) are emboldened

Participants with migraine reported headache on an average of 3.9 days/month with a mean duration of 11.1 h and a mean intensity of 1.7 (moderate). Mean pTIS was 5.3% (Table 1). Applying the DW for migraine (0.441 [21]), we estimated mean lost health as 2.3%. pTIS was higher among females than males (6.2% vs. 3.7%; *p* < 0.001), the result both of higher frequency (4.2 vs. 3.3 days/month; *p* = 0.002) and of longer duration (12.5 vs. 8.8 h; *p* < 0.001). Intensity did not differ between genders. Impaired participation with migraine was reflected in 0.9 lost days/3 months from paid work, 2.0 days from household work (with no differences between genders) and 0.4 days from social or leisure activities (males [0.5] more than females [0.3]; *p* = 0.03) (Table 2).

Participants with TTH reported headache on an average of 2.7 days/month with a mean duration of 4.6 h and a mean intensity of 1.1 (mild). Mean pTIS was 1.5%. With DW for TTH = 0.037 [21], mean lost health at individual level was 0.1%, at the lower limit of measurement. There were no differences in these measures between genders (Table 1). TTH caused little impairment of participation (< 1 day/3 months in any domain [Table 2]), but males lost more days from paid work (0.7) than females (0.2; *p* < 0.001) (Table 2).

Participants with pMOH reported headache on an average of 22.2 days/month with a mean duration of 7.4 h and a mean intensity of 2.0 (moderate). Mean pTIS was 24.9%, and mean lost health was 5.6% (DW for MOH = 0.223 [21]). None of these differed between genders (Table 1). Impaired participation was reflected in 4.5 days/3 months lost from paid work, 5.6 days from household work and 2.0 days from social or leisure activities (Table 2). Males (10.2 days/3 months) reported much higher losses from paid work than females (1.1 days; *p* = 0.01) (Table 2).

Participants with other H15 + reported headache on an average of 17.8 days/month with a mean duration of 7.6 h and a mean intensity of 1.8 (moderate). Mean pTIS was 14.9%. Impaired participation was reflected in 2.3 days/3 months lost from paid work (males [4.0] more than females [1.2]; *p* = 0.01), 4.5 days from household work and 0.7 days from social or leisure activities (Table 2).

Headache type had a significant (*p* < 0.001) influence on impaired participation in all domains (Table 2; Fig. 1). H15+ (pMOH or other) was associated with significantly greater losses from paid and household work than migraine or TTH, and migraine with greater losses than TTH (non-overlapping 95% CIs in Fig. 1). Differences in social or leisure activities were smaller but still significant.

### Headache yesterday

Table 3 shows the symptom burden and impaired participation yesterday in the 12.1% of participants reporting HY. Mean duration was 3.9 h, mean intensity 1.5 (mild-to-moderate). Of everyone with HY, only 26.3% could do everything as normal or more than half, so that almost three quarters (73.7%) could do less than half or nothing. There were no significant differences between genders (Table 3).

**Table 3:** Headache yesterday burden and impaired participation at individual level, by gender

	Overall	Male	Female	Male vs. female
**Duration** (hours)				
mean±SEM median	3.9±0.3 2.0	3.4±0.3 2.0	4.3±0.4 3.0	F(1, 258) = 3.1 *p* = 0.08
**Intensity**				
mild (n) (%) moderate (n) (%) severe (n) (%) mean*	156 (60.0) 89 (34.2) 15 (5.8) 1.5	61 (59.8) 34 (33.3) 7 (6.9) 1.5	95 (60.1) 55 (34.8) 8 (5.1) 1.5	*X*^*2*^(2, 260) = 0.4 *p* = 0.82
**What done**				
everything (n) (%) more than half (n) (%) less than half (n) (%) nothing (n) (%)	16 (6.1) 53 (20.2) 76 (29.8) 115 (43.9)	6 (5.0) 23 (19.0) 34 (28.1) 58 (47.9)	10 (7.2) 30 (21.6) 42 (30.2) 57 (41.0)	X2(3, 260) = 1.5 *p* = 0.67

* Equating to 1, 2, 3 and treating as though continuous data

**Table 4:** Willingness to pay for effective treatment, and self-reported quality of life in people with headache and those without

Headache status	Willingness to pay (PEN/month)	Quality of life	
	mean±SEM, median	mean±SEM, median	p (vs. no headache)
pMOH	72.5±39.3, 40.0	25.0±0.7, 26.0	**0.001**
Other H15+	46.6±8.9, 25.0	25.3±0.4, 25.0	**< 0.001**
Migraine	35.3±5.3, 20.0	26.1±0.1, 26.0	**< 0.001**
TTH	27.7±1.8, 20.0	27.0±0.1, 27.0	0.36
No headache	-	27.2±0.1, 28.0	-
	F(3, 611) = 3.2 ***p*** **= 0.02**	F(4, 2144) = 14.6 ***p*** **< 0.001**	-

PEN: Peruvian nuevo sol (at the time of the study, PEN 1.00 = USD 0.30); pMOH: probable medication-overuse headache; H15+: headache on ≥ 15 days/month; TTH: tension-type headache; significant p-values (< 0.05) are emboldened

**Table 5:** **Proportion of time in ictal state and impaired participation at population level**,** by headache type and by timeframe of enquiry** (adjusted for age and gender)

Headache type	Estimated pTIS (%)		Estimated impaired participation			
	According to 1-year prevalence and reported average frequency and usual duration	According to prevalence and duration of headache yesterday	According to HALT data (lost days/3 months)			According to headache yesterday
			**Lost productivity**		**Social or leisure activities**	**Total impaired participation** **(%)**
			**Paid work**	**Household work**		
All headache	2.5	1.9	0.5	1.0	0.2	8.7
pMOH	0.2		0.0	0.1	0.0	
Other H15+	0.5		0.1	0.1	0.0	
Migraine	1.2		0.2	0.5	0.1	
TTH	0.6		0.2	0.3	0.1	

pTIS: proportion of time in ictal state; HALT: Headache-Attributed Lost Time index; H15+: pMOH: probable medication-overuse headache; headache on ≥ 15 days/month; TTH: tension-type headache

### Willingness to pay and quality of life

WTP differed significantly between headache types (*p* = 0.02), with clear gradation from TTH (least) through migraine and other H15 + to pMOH (most) (Table 4). The differences between means and medians were indicative of skewed data (minorities willing to pay substantially more).

**Fig. 2:**
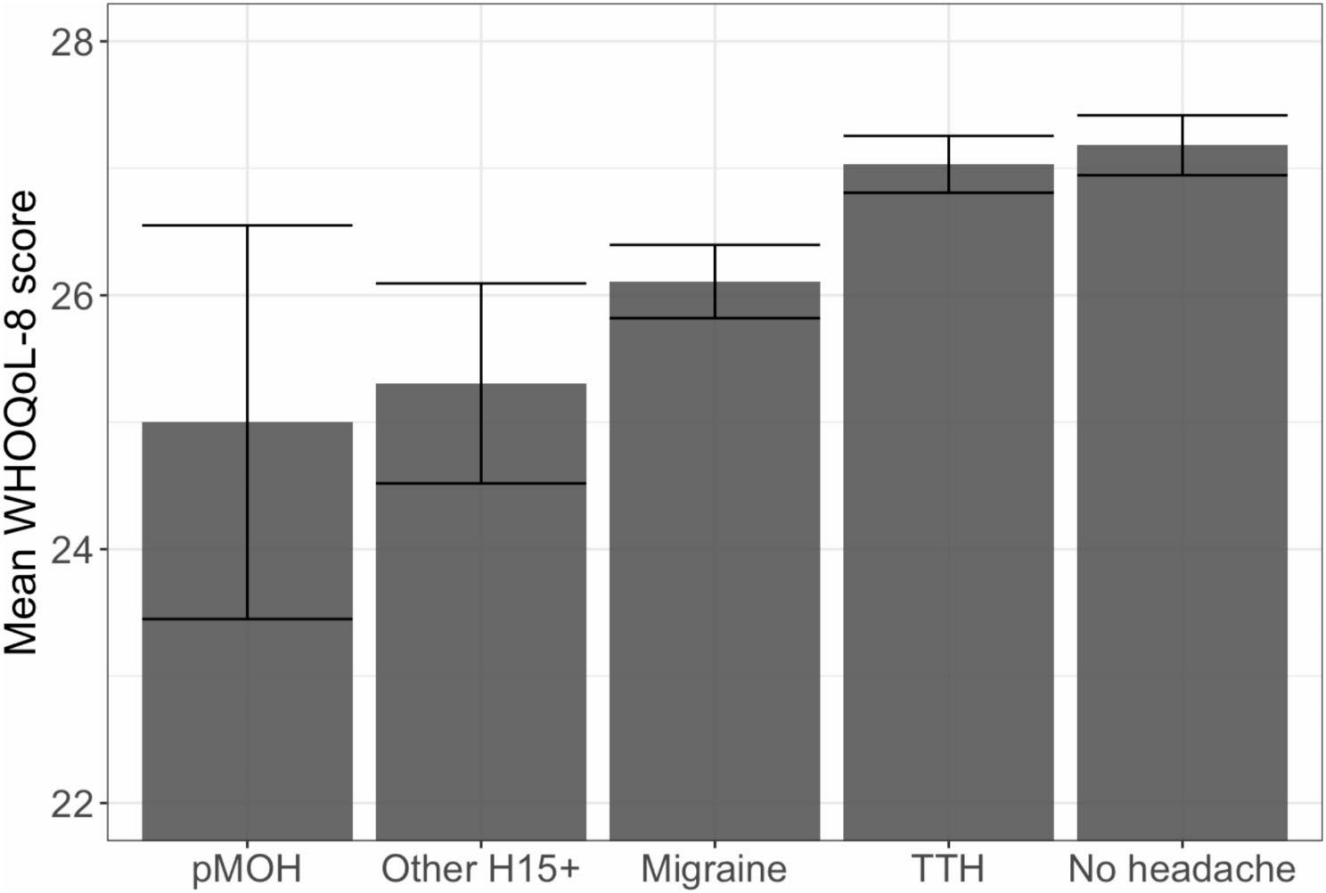
Quality of life (measured with WHOQoL-8) by headache status (pMOH: probable medication-overuse headache; H15+: headache on ≥ 15 days/month; TTH: tension-type headache; error bars are 95% confidence intervals)

All headache types except TTH impacted QoL, with a similar gradation: TTH (least) through migraine and other H15 + to pMOH (most) (Table 4; Fig. 2).

## Population-level estimates

Table 5 shows the age- and gender-adjusted estimates of pTIS and impaired participation at population level. Based on the 1-year prevalence and reported frequency and usual duration of headache, population-level pTIS for all headache was 2.5%. About half (1.2%) was with migraine, while TTH (0.6%), pMOH (0.2%) and other H15+ (0.5%) contributed less. Using HY data, we estimated population-level pTIS for all headache to be lower, at 1.9%.

Population-level estimates of mean lost health attributed to each headache type were 0.5% for migraine and 0.0% (i.e., below the limit of estimation) for TTH and MOH.

**Table 6:** Health-care needs assessment

Criterion fulfilled		Proportion of sample		Estimated proportion of adult population* % [95% CI]
		*n*	%	
1	Headache on ≥ 15 days/month	96	4.5	3.9 [3.1–4.8]
2	Migraine on ≥ 3 days/month	291	13.5	13.7 [12.3–15.2]
3	Migraine and pTIS > 3.3% and moderate-to-severe intensity	157^1^	7.3	7.4 [6.3–8.6]
4	Migraine and lost work and/or household days/3 months ≥ 3	151^2^	7.0	7.7 [6.6–8.9]
5	TTH and pTIS > 3.3% and moderate-to-severe intensity	20	0.9	0.9 [0.5–1.4]
6	TTH and lost work and/or household days/3 months ≥ 3	130^3^	6.0	6.1 [5.1–7.2]
One or more of criteria 1–6		590	27.5	27.8 [25.9–29.8]

*Age- and gender-corrected; ^1^of whom 147 also fulfilled criterion 2; ^2^of whom 99 also fulfilled criterion 2, 56 also fulfilled criterion 3 and 51 also fulfilled criteria 2 and 3; ^3^of whom 4 also fulfilled criterion 5; pTIS: proportion of time in ictal state; TTH: tension-type headache

Impaired participation at population level for all headache was estimated from HALT data (90-day recall) at 0.5 days/3 months lost from paid work, 1.0 days from household work 0.2 days from social or leisure activities. Migraine again contributed half (0.2, 0.5 and 0.1 days/3 months), TTH rather less (0.2, 0.3 and 0.1 days/3 months) and pMOH and other H15 + relatively little (Table 5). HY data indicated overall impairment of participation, across all domains, of 8.7%.

### Health-care needs assessment

Table 6 shows numbers and proportions of participants fulfilling each of the health-care needs criteria, with 590 (27.5%) fulfilling one or more and therefore presumed to be in need of care. Adjusted for age and gender, this translated into 27.8% of the adult proportion aged 18–65 years, the majority having migraine (16.9%) and much lesser proportions having TTH (6.8%) or H15+ (3.9%).

## Discussion

This was the first study evaluating the burden of headache disorders in the adult general population of Peru. It used the standardized methodology developed by the Global Campaign against Headache [3, 4] and previously applied in multiple other studies [5–17]. We found high levels of burden at both individual and population levels. Employing two different methodologies, we estimate that 1.9–2.5% of all time among adults in Peru is spent with headache, migraine (1.2%) being the biggest contributor.

At individual level, H15 + was, of course, associated with greater symptom burden than either migraine or TTH. This was reflected in mean pTIS estimates of 24.9% for pMOH and 14.9% for other H15 + compared with 5.3% for migraine and only 1.5% for TTH. It was reflected in impaired participation in both paid and unpaid work: 4.5 and 5.6 lost days/3 months respectively for pMOH, 2.3 and 4.5 days for other H15+. And it was reflected in WTP and QoL, both subjective measures of overall burden and both showing gradation from pMOH (greatest) through other H15 + and migraine to TTH (least).

This suggests H15 + should have highest priority for health care, especially pMOH, effective treatment (drug withdrawal) of which is cheap and can greatly improve the lives of those who have developed this disorder [24, 25]. On the other hand, good care for migraine (especially) and TTH should pre-empt development of most pMOH. Further, at population level, migraine was the main contributor not only to pTIS (1.2% out of 2.5% total) but also to impaired participation (0.2 out of 0.5 total lost days/3 months from paid work, 0.5 out of 1.0 days from household work), followed in both cases by TTH (0.6%; 0.2 and 0.3 days). Headache services need to embrace all these headache types.

This brings us to the needs assessment. The high population-level burden attributed to migraine was a consequence of its prevalence (22.8%) along with its high individual burden. The lesser but still high population-level burden attributed to TTH was driven mainly by its very high prevalence (38.9%), with lower individual burden. pMOH (1.2%) and other H15+ (2.7%) were much less prevalent [17]. It is no surprise that the majority of those fulfilling our health-care needs criteria had migraine.

In total, over one quarter (27.5%) of the adult population (aged 18–65 years) of Peru was estimated to need headache care: that is, they were assessed as being likely to benefit from professional care. This is a costly requirement. But the totals of 0.5 lost days/3 months from paid work and 1.0 days from household work represent lost productivity. The former may translate into a loss of 0.8% (0.5/65) from gross domestic product (GDP), assuming a 5-day working week. In other words, untreated or ineffectively treated headache is also costly. Taking this into account, headache services reaching all who need them are expected to be cost-effective, and potentially cost saving [26].

But, as we have previously discussed [27], there is no certain way with HALT data to translate days lost into proportions because the denominators (days/3 months of expected work) for each participant are not known: in particular, not everybody has a 5-day working week (many may not be engaged in paid or income-generating work at all), and days of expected household work are highly variable. Differences between genders are likely, and these are illustrated in the individual data shown in Table 2: males reported more lost time from paid work than females, and females reported more lost time from household work than from paid work, whereas males did not. (It should be remembered that household work means the essential chores of daily life, not housekeeping.)

Estimates based on HY, however, do provide denominators, since the enquiry related to a single specific day, and responses would have related to whatever had been planned for that day. They also have the benefit of minimal recall error, unlike HALT data recalled over 90 days. Estimates based on HY indicated that 8.7% of all activity (work and non-work) among the adult population of Peru is lost to headache, a greater burden than is implied by 0.5 lost days/3 months from paid work, 1.0 days from household work and 0.2 days from social or leisure activities. If, for example, we assume mean denominators per 3 months of 32.5 (2.5 days/week) for both paid and household work, and 26 (2 days/week) for social or leisure activities, estimated population-level impaired participation based on HALT is 5.4% ([0.5/32.5]+[1.0/32.5]+[0.2/26]*100%). However, impaired participation of 8.7% estimated from HY is very much greater than the population-level pTIS of 1.9% also estimated from HY, a strong indication that participation is impaired beyond the time during which headache is present. The reported mean duration of HY was rather short (3.9 h), but we have previously shown that merely having headache on a particular day leads to productivity loss that day, regardless of its duration [28]. It is not surprising if a person with headache for most of the morning abandons planned activity for the day.

The importance is that headache-attributed impaired participation – a key factor in the economics of headache and its effective treatment – is not reliably recalled over periods of 3 months. Given this, and the disparity between recalled and HY data, productivity losses – including losses to GDP – may be substantially greater than are indicated by the former.

There are no other data from South America with which to make comparisons. With regard to the headache-care needs assessment, Peru (27.5% of the population aged 18–65 years) is towards the lower end of the range established by the four other studies that used the same or very similar methodology: Cameroon 37.0% [29], Saudi Arabia 35.8% [27], Mongolia 33.2% [30] and Mali (with slightly modified criteria) 23.4% [31].

### Strengths and limitations

Strengths were the use of standardized methodology [3, 4], an adequately sized sample (*N* = 2,149) [2] drawn from five geographical regions to ensure national representativeness, adjustment of population-level estimates for age and gender, and collection of data relating to the preceding day to complement those dependent on recall.

There were some limitations, including those inherent in cross-sectional studies, which have been described previously [17]. We did not validate the diagnostic questions within the sample, but the same question set has been validated in six previous studies [32–37]. The questionnaire allowed only one diagnosis per participant, which may have led to underestimation of TTH-attributed burden in those with both migraine and TTH (migraine usually being more bothersome).

## Conclusion

Headache disorders are not only prevalent in Peru but also lead to high levels of ill health and economic burden in Peru, with a substantial requirement for health care. Migraine is responsible for the largest share of these at population level, but health services for headache should recognize the needs of those with H15+, and of many with TTH, who have high individual burden. Health and economic policies balancing the health benefits against the cost of providing care should take account of the productivity losses that might be recovered, and the expected cost-offset.

## Data Availability

The original data are held on file at the Peruvian University Cayetano Heredia, Lima, Peru, and the analytical dataset at Norwegian University of Science and Technology. Once analysis and publications are completed, they will be freely available for non-commercial purposes to any person requesting access in accordance with the general policy of the Global Campaign against Headache.

## Abbreviations

ANOVA: Analysis of variance
aOR: Adjusted odds ratio
CI: Confidence interval
d/m: Days/month
DW: Disability weight
GBD: Global Burden of Disease (study)
GDP: Gross domestic product
HALT: Headache-Attributed Lost Time (index)
HARDSHIP: Headache-Attributed Restriction, Disability, Social Handicap and Impaired Participation (questionnaire)
ICHD: International Classification of Headache Disorders
HY: Headache yesterday
MOH: Medication-overuse headache
OR: Odds ratio
PEN: Peruvian nuevo sol
pMOH: Probable MOH
pTIS: Proportion of time in ictal state
QoL: Quality of life
SEM: Standard error of the mean
TTH: Tension-type headache
USD: United States dollar
WHOQoL-8: World Health Organization Quality of Life (8-item questionnaire)
